# The Lung Inflammation and Skeletal Muscle Wasting Induced by Subchronic Cigarette Smoke Exposure Are Not Altered by a High-Fat Diet in Mice

**DOI:** 10.1371/journal.pone.0080471

**Published:** 2013-11-19

**Authors:** Michelle J. Hansen, Hui Chen, Jessica E. Jones, Shenna Y. Langenbach, Ross Vlahos, Rosa C. Gualano, Margaret J. Morris, Gary P. Anderson

**Affiliations:** 1 Lung Health Research Centre, Department of Pharmacology and Therapeutics, The University of Melbourne, Victoria, Australia; 2 Department of Pharmacology, School of Medical Sciences, University of New South Wales, Sydney, New South Wales, Australia; 3 School of Medical and Molecular Biosciences, Faculty of Science, University of Technology, Sydney, New South Wales, Australia; National Heart and Lung Institute, United Kingdom; Ito

## Abstract

Obesity and cigarette smoking independently constitute major preventable causes of morbidity and mortality and obesity is known to worsen lung inflammation in asthma. Paradoxically, higher body mass index (BMI) is associated with reduced mortality in smoking induced COPD whereas low BMI increases mortality risk. To date, no study has investigated the effect of a dietary-induced obesity and cigarette smoke exposure on the lung inflammation and loss of skeletal muscle mass in mice. Male BALB/c mice were exposed to 4 cigarettes/day, 6 days/week for 7 weeks, or sham handled. Mice consumed either standard laboratory chow (3.5 kcal/g, 12% fat) or a high fat diet (HFD, 4.3 kcal/g, 32% fat). Mice exposed to cigarette smoke for 7 weeks had significantly more inflammatory cells in the BALF (P<0.05) and the mRNA expression of pro-inflammatory cytokines and chemokines was significantly increased (P<0.05); HFD had no effect on these parameters. Sham- and smoke-exposed mice consuming the HFD were significantly heavier than chow fed animals (12 and 13%, respectively; P<0.05). Conversely, chow and HFD fed mice exposed to cigarette smoke weighed 16 and 15% less, respectively, compared to sham animals (P<0.05). The skeletal muscles (soleus, tibialis anterior and gastrocnemius) of cigarette smoke-exposed mice weighed significantly less than sham-exposed mice (P<0.05) and the HFD had no protective effect. For the first time we report that cigarette smoke exposure significantly decreased insulin-like growth factor-1 (IGF-1) mRNA expression in the gastrocnemius and tibialis anterior and IGF-1 protein in the gastrocnemius (P<0.05). We have also shown that cigarette smoke exposure reduced circulating IGF-1 levels. IL-6 mRNA expression was significantly elevated in all three skeletal muscles of chow fed smoke-exposed mice (P<0.05). In conclusion, these findings suggest that a down-regulation in local IGF-1 may be responsible for the loss of skeletal muscle mass following cigarette smoke exposure in mice.

## Introduction

Globally tobacco smoking and obesity are two common causes of preventable morbidity and mortality. Tobacco smoking is the leading preventable cause of death in adults and accounted for more than five million deaths worldwide in 2005 [1]. Smoking is the major cause of chronic obstructive pulmonary disease (COPD), a progressive disease characterized by irreversible airflow limitation and lung inflammation, which is the fourth leading cause of death worldwide [2]. In 2008, more than 1.4 billion adults worldwide were classified as overweight or obese [3]. Being overweight and obese constitutes major risk factors for type 2 diabetes, cardiovascular disease and certain forms of cancer. While obesity is a complex multifactorial disease, the dramatic increase in prevalence most likely reflects increased consumption of energy rich foods and decreased physical activity [3]. Obesity has been shown to worsen systemic inflammation and disease in a number of clinical studies and experimental models [4]–[7]. Paradoxically, a higher rather than lower body mass index (BMI) is associated with better long-term outcomes in COPD [8], [9].

While the effects of tobacco smoking on the respiratory tract are well established, systemic consequences such as cardiovascular disease [10], atrophy of skeletal muscle fibers [11] and muscle dysfunction [12] are becoming increasingly recognized as contributors to morbidity. While the decline in lung health is directly caused by tobacco smoke, the pathophysiological mechanisms responsible for the systemic effects remain poorly understood. A number of population based studies have confirmed a state of chronic low-grade systemic inflammation, including elevated serum levels of tumor necrosis factor-α (TNF-α, interleukin-6 (IL-6) and C-reactive protein (CRP), in smokers [10]. Moreover, in COPD elevated circulating levels of TNF-α, IL-6, and CRP have been associated with skeletal muscle loss and reduced exercise capacity [13]-[15]. In rodents, administration of pro-inflammatory cytokines such as IL-1β, IL-6 and TNF-α induce a severe wasting syndrome [16], [17]. The pro-inflammatory cytokines TNF-α and IL-6 can induce muscle wasting by activating key mediators in the ubiquitin-proteasome pathway [18], [19]. TNF-α also inhibits skeletal muscle regenerative pathways through the induction of oxidative stress [20] and via NFκB-dependent inhibition of MyoD, the transcription factor essential for regeneration of muscle [21], [22].

A number of experimental models of wasting have identified the importance of the ubiquitin-proteasome pathway [23], [24]. The muscle specific E3 ligases, muscle-specific ring finger 1 (MuRF1) and atrogin-1 (also known as muscle atrophy Fbox protein), are induced in a number of rodent models of skeletal muscle wasting including cancer, diabetes, denervation and disuse [24]. Alternatively, mice deficient in either atrogin-1 or MuRF1 [23] were protected against skeletal muscle wasting. Insulin-like growth factor 1 (IGF-1) decreases the activity of the ubiquitin-proteasome pathway by inhibiting the transcription of atrogin-1 and MuRF1 [25]. In addition, IGF-1 promotes skeletal muscle hypertrophy by increasing protein synthesis and satellite cell proliferation and differentiation [26], [27], thus states of low IGF-1 may promote skeletal muscle wasting.

While current smokers tend to have a lower BMI, central or abdominal obesity appears to be increased and this is associated with adverse metabolic consequences [28]. Thus the weight loss associated with tobacco smoking may be due to loss of lean mass rather than fat. Although obesity may be protective in patients with severe COPD [29], obesity and overweight and tobacco smoking are associated with low grade systemic inflammation, thus increased body weight induced by a palatable high fat diet (HFD) may alter the skeletal muscle wasting induced by cigarette smoke exposure in mice. To examine this we used well established models of diet-induced obesity [30], [31] and cigarette smoke exposure [32], [33] and examined parameters of lung inflammation, body weight and skeletal muscle wasting where we have previously documented the effect smoke exposure and HFD on the hypothalamic appetite regulator neuropeptide Y and fat accumulation [33]. We have measured a number of pro-inflammatory cytokines (IL-1β, IL-6, TNF-α), chemokines (MCP-1, MIP-2 and KC) and a macrophage growth factor GM-CSF as they have been shown to be important in cigarette smoke-induced lung inflammation [34]. We measured the expression of genes that induce skeletal muscle wasting such as atrogin-1 and MuRF1. As the anabolic hormone IGF-1 inhibits the induction of these factors [25], [35] we measured skeletal muscle gene and protein expression as well as plasma concentration of IGF-1. Furthermore, as low grade systemic inflammation has been implicated in skeletal muscle wasting we measured plasma concentrations of IL-6 and serum amyloid A (SAA; a pro-inflammatory acute phase protein induced in the liver by circulating cytokines IL-1β, IL-6 and TNF-α) as well as TNF-α and IL-6 gene expression and protein in skeletal muscle.

This is the first study to examine the effect of a HFD on the lung inflammation and skeletal muscle loss induced by cigarette smoke exposure in mice and identifies an important role for IGF-1 in the skeletal muscle loss induced by cigarette smoke exposure in mice.

## Materials and Methods

### Animals

Specific pathogen-free male BALB/c mice were obtained from the Animal Resource Centre Pty. Ltd. (Perth, WA, Australia), housed at 20±2°C in sterile micro-isolator cages, and maintained on a 12:12 h light/dark cycle (lights on at 06:00 h). Mice were allowed to acclimatize to their new environment for one week, with *ad libitum* access to sterile standard laboratory chow. All procedures were approved by the Animal Experimentation Ethics Committee of the University of Melbourne.

### Treatment

After acclimatization, mice were randomly divided into four groups (n = 8 per group) that were matched for body weight. Two groups of animals were exposed to cigarette smoke and two groups were sham exposed according to our published protocol [32], [34]. Briefly, animals underwent whole-body exposure to the smoke of 2 filtered cigarettes (Winfield Red, 16 mg≤of tar, 1.2 mg≤of nicotine and 15 mg≤of CO, Philip Morris, Melbourne, Australia), twice a day (10:30 h and 16:30 h), 6 days a week for 7 weeks inside an 18 liter plastic chamber. Sham animals were handled identically without cigarette smoke exposure. Animals consumed either standard laboratory chow (chow; 3.54 kcal/g, fat 12%, protein 22%, carbohydrate 66%) or a high-fat diet (HFD; 4.32 kcal/g, fat 32% (saturated fat 17%), protein 18%, carbohydrate 50%). This well characterized [30], [36] HFD consists of modified laboratory chow containing sweetened condensed milk and lard, supplemented with highly palatable cafeteria style foods such as meat pies, cakes, and biscuits. Thus the four groups were 1) sham and chow fed, 2) sham and HFD fed, 3) smoke exposed and chow fed and 4) smoke exposed and HFD fed.

### Tissue Collection

After the seven week protocol mice were given an anesthetic overdose (ketamine and xylazine, 180 and 32 mg/kg i.p., respectively), and blood was collected from the abdominal vena cava and processed for plasma. Separated plasma was stored at −80°C for subsequent determination of plasma IGF-I, cytokine and SAA concentrations. Whole lungs were perfused free of blood via right ventricular perfusion with 10 ml of PBS, rapidly removed, blotted and snap frozen in liquid nitrogen and stored at −80°C for subsequent quantitative RT-PCR and protein determination by ELISA. The gastrocnemius, soleus and tibialis anterior hind limb skeletal muscles were weighed and snap frozen in liquid nitrogen and stored at −80°C for subsequent analysis. The reported weights are the average of the left and right skeletal muscle.

### Cellular Inflammatory Response

Bronchoalveolar lavage fluid (BALF) was collected as previously described [34]. Viable cells in BALF were counted by fluorescence microscopy and cytospins were prepared using 200 µl BALF spun at 350 rpm for 10 min on a Cytospin 3 (Shandon, UK). Cytospin slides were stained with DiffQuik (Dade Baxter, Australia) and 500 cells per slide were differentiated into eosinophils, neutrophils, lymphocytes and macrophages by standard morphological criteria. BALF was centrifuged to pellet cells and the clarified BALF stored at −80°C.

### Preparation of tissue homogenates for protein determination

As previously described [37], [38], an amount of frozen lung tissue or gastrocnemius skeletal muscle was weighed and placed into a tube containing T-PER tissue protein extraction reagent (Thermo Fisher Scientific, IL, USA) and Halt Protease Inhibitor Cocktail (1∶100 dilution; Thermo Fisher Scientific). The tissues were homogenized using the Qiagen TissueLyser (MD, USA) and Qiagen stainless steel beads (5 mm). The homogenates were centrifuged at 9 000 g for 10 minutes at 4°C and the supernatants collected and frozen at −80 for subsequent analysis using ELISA. Total protein concentration in lung was determined using the BCA protein assay method (Bio-Rad, CA, USA).

### ELISA

The protein concentrations of GM-CSF, IL-1β, IL-6, IGF-I, KC, MCP-1, MIP-2, SAA and TNF-α, were determined using commercially available ELISA kits. R&D Systems (MN, USA) DuoSet ELISA kits were used to measure IL-1β IGF-I, KC, TNF-α, and MIP-2 levels, and eBioscience ELISA kits (CA, USA) were used to determine IL-6, GM-CSF and MCP-1 levels. Plasma SAA concentration was determined using an ELISA kit from BioSource (CA, USA).

### Quantitative RT-PCR

Total RNA was isolated from lung tissue and skeletal muscle using an RNeasy kit (Qiagen, MD, USA) according to the manufacturer's instructions. The purified total RNA was used as a template to generate first-strand cDNA synthesis using SuperScript III (Invitrogen, CA, USA). Pre-optimized TaqMan® gene expression assays (Applied Biosystems, CA, USA) were used for RT-PCR using an ABI 7900 HT Sequence Detection System (Applied Biosystems). Gene expression was quantified in a single multiplexing reaction, where our gene of interest (for skeletal muscle: IGF-I, atrogin-1, MuRF1, IL-6, and TNF-α; for lung: IL-1β, IL-6, KC, TNF-α, MCP-1, MIP-2, and GM-CSF) was standardized to 18S rRNA as previously described [34]. For each gene of interest an individual sample from the sham and chow fed group was assigned as a calibrator against which all other samples are expressed as a fold difference.

### Statistical analyses

Results are expressed as mean±SE. All data were analyzed using two-way ANOVA and when statistical significance was achieved a *post hoc* Bonferroni test was used to compare between treatment groups. All statistical analyses were performed with GraphPad Prism for Windows (version 4.02). In all cases, probability values less than 0.05 (P<0.05) were considered statistically significant.

## Results

### Effect of cigarette smoke exposure and HFD on BALF cellularity

**Figure 1:**
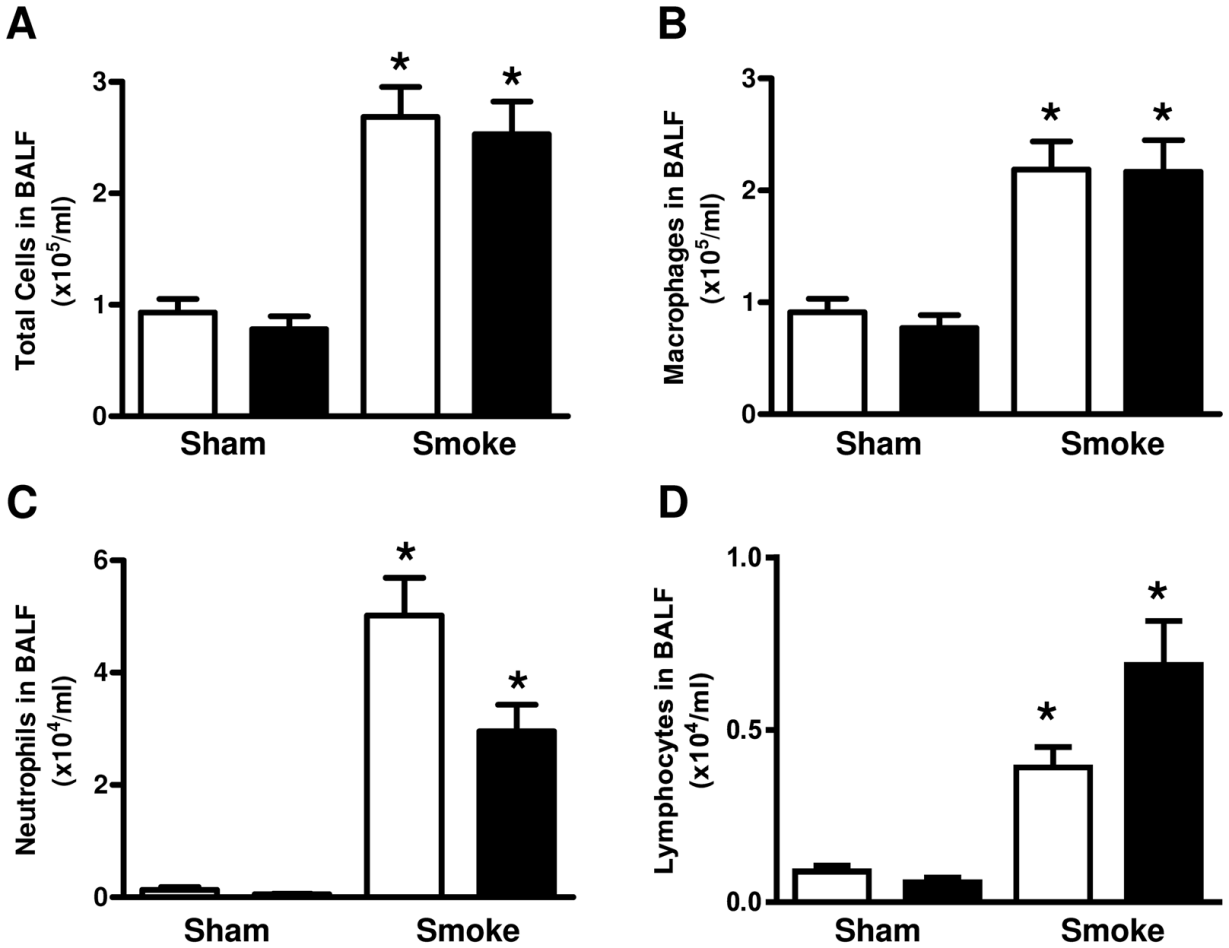
Effect of subchronic cigarette smoke exposure and HFD on BALF cellularity. Male BALB/c mice were exposed to 4 cigarettes/day, 6 days/week for 7 weeks and the number of total cells (A), macrophages (B), neutrophils (C) and lymphocytes (D) were counted. Mice had access to either standard laboratory chow (□) or a HFD (▪) across the 7 week experimental period. Data are shown as mean±SE for n = 8 per treatment group. Data were analysed by two-way ANOVA and when significance was achieved a *post hoc* Bonferroni test was performed. * P<0.05 significant *post hoc* effect of smoke exposure compared to sham animals on the same diet.

Cigarette smoke exposure (4 cigarettes/day, 6 days/week for 7 weeks) significantly increased the total, macrophage, neutrophil and lymphocyte number in BALF (P<0.05, Figure 1A-D). The HFD had no significant effect on BALF cellularity in either the sham or smoke-exposed animals (Figure 1A-D).

### Effect of cigarette smoke exposure and HFD on cytokine and chemokine protein levels in BALF

Cigarette smoke exposure significantly elevated GM-CSF and KC protein concentrations in BALF (P<0.05, Table 1). There was no effect of the HFD or cigarette smoke exposure on IL-1β, IL-6, TNF-α or MIP-2, protein levels in BALF. MCP-1 was not detectable in BALF.

**Table 1:** The effect of subchronic cigarette smoke exposure and a HFD on BALF protein levels of cytokines and chemokines.

	Sham		Smoke	
	Chow	HFD	Chow	HFD
IL-1β protein (pg/ml)	60±10	51±11	74±10	61±12
IL-6 protein (pg/ml)	225±29	175±23	164±26	187±29
TNF-α protein (pg/ml)	147±26	132±32	142±24	171±25
MIP-2 protein (pg/ml)	160±19	148±18	166±20	181±18
KC protein (pg/ml)	43±14	23±3	255±39*	235±31*
GM-CSF protein (pg/ml)	58±18	39±20	164±13*	163±33*

MCP-1 protein concentration in BALF was below the detection sensitivity of the ELISA.

Results are expressed as mean±SE; n = 8 per group. Data were analysed by two-way ANOVA and where appropriate a *post hoc* Bonferroni test was used.

* P<0.05 significant *post hoc* effect of smoke exposure compared to sham animals on the same diet.

### Effect of cigarette smoke exposure and HFD on cytokine and chemokine gene and protein levels in lung tissue

Cigarette smoke exposure significantly elevated the mRNA expression of IL-1β, IL-6, MCP-1, MIP-2, KC and GM-CSF in the lung tissue of chow and HFD fed animals (Table 2, P<0.05). While cigarette smoke exposure had a significant effect on TNF-α mRNA expression (P = 0.02) in the lung tissue of chow and HFD fed animals this did not reach statistical significance following *post-hoc* analysis. There was no effect of the HFD or cigarette smoke exposure on protein levels of these cytokines and chemokines in lung tissue (data not shown). The HFD did not significantly alter the expression of the genes examined (Table 2).

**Table 2:** The effect of subchronic cigarette smoke exposure and a HFD on lung tissue mRNA expression of cytokines and chemokines.

	Sham		Smoke	
	Chow	HFD	Chow	HFD
IL-1β mRNA (fold 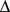 )	1.02±0.06	1.06±0.09	1.98±0.15*	2.08±0.25*
IL-6 mRNA (fold 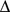 )	1.00±0.11	1.18±0.16	2.37±0.22*	3.06±0.66*
TNF-α mRNA (fold 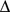 )	1.00±0.21	0.81±0.07	2.20±0.58	2.01±0.54
MCP-1 mRNA (fold 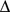 )	1.00±0.15	0.93±0.16	3.51±0.62*	3.51±0.50*
MIP-2 mRNA (fold 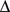 )	1.00±0.12	0.73±0.05	4.74±0.51*	4.94±0.54*
KC mRNA (fold 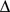 )	1.05±0.13	0.87±0.09	12.98±1.29*	15.92±1.56*
GM-CSF mRNA (fold 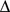 )	1.00±0.10	1.07±0.07	2.87±0.39*	3.07±0.27*

Results are expressed as mean±SE; n = 8 per group. Data were analysed by two-way ANOVA and where appropriate a *post hoc* Bonferroni test was used.

* P<0.05 significant *post hoc* effect of smoke exposure compared to sham animals on the same diet.

### Effect of cigarette smoke exposure and HFD on body weight, skeletal muscle weights and plasma IGF-I and SAA concentrations

After 7 weeks of consuming the HFD, the body weight of the sham and cigarette smoke exposed animals was significantly increased compared to the appropriate chow fed group by 12% and 13%, respectively (Table 2, P<0.05). Conversely, mice exposed to cigarette smoke had significantly lower body weight compared to the sham mice (chow fed animals by 16% and HFD fed animals by 15%; Table 3, P<0.05).

**Table 3:** The effect of subchronic cigarette smoke exposure and a HFD on body weight, skeletal muscle weights and circulating IGF-I and SAA protein levels.

	Sham		Smoke	
	Chow	HFD	Chow	HFD
Body weight (g)	26.6±0.4	29.8±0.7†	22.4±0.5*	25.3±0.4* †
Soleus skeletal muscle (mg)	7.1±0.1	7.8±0.1†	6.4±0.6*	6.8±0.1*
Tibialis anterior skeletal muscle (mg)	51.7±0.9	53.1±1.0	46.4±1.5*	47.1±0.4*
Gastrocnemius skeletal muscle (mg)	128.8±1.6	132.7±2.5	116.1±3.8*	119.8±1.1*
Plasma IGF-I (ng/ml)	117.1±7.7	139.0±11.4	97.7±6.6	103.4±6.1*
Plasma SAA (μg/ml)	8.2±0.5	8.6±0.4	9.1±0.7	9.7±1.3

Results are expressed as mean±SE; n = 8 per group. Data were analysed by two-way ANOVA and where appropriate a *post hoc* Bonferroni test was used.

* P<0.05 significant *post hoc* effect of smoke exposure compared to sham animals on the same diet.

† P<0.05, significant *post hoc* effect of the HFD compared to chow fed animals.

Cigarette smoke exposure significantly decreased the weights of all three skeletal muscles examined in both dietary groups (Table 3, P<0.05). Specifically, cigarette smoke exposure significantly reduced the weights of the soleus, tibialis anterior and gastrocnemius skeletal muscles by 9.2, 10.3 and 9.9%, respectively, in the chow fed animals and by 12.6, 11.3 and 9.7%, respectively, in the HFD fed animals. The HFD had no effect on the tibialis anterior and gastrocnemius skeletal muscle weights for the sham and cigarette smoke exposed animals. The HFD significantly increased the weight of the soleus skeletal muscle of the sham animals by 9.9% compared to those consuming chow (P<0.05).

Cigarette smoke exposure had a significant effect on the plasma concentration of the anabolic hormone IGF-1 and *post hoc* analysis revealed a significant effect in the HFD fed animals with smoke exposure decreasing plasma IGF-1 level by 26% compared to sham HFD fed animals (Table 3, P<0.05). Plasma IGF-1 level was not significantly altered by the HFD (Table 3). In the current study, while there was an induction of inflammatory genes in the lung following cigarette smoke exposure systemic inflammation as measured by SAA was not significantly altered. Specifically, both the HFD and cigarette smoke exposure had no significant effect on plasma SAA concentration (Table 3). Plasma concentration of IL-6 was below the level of detection of the ELISA used.

### Effect of cigarette smoke exposure and HFD on skeletal muscle gene expression

**Figure 2:**
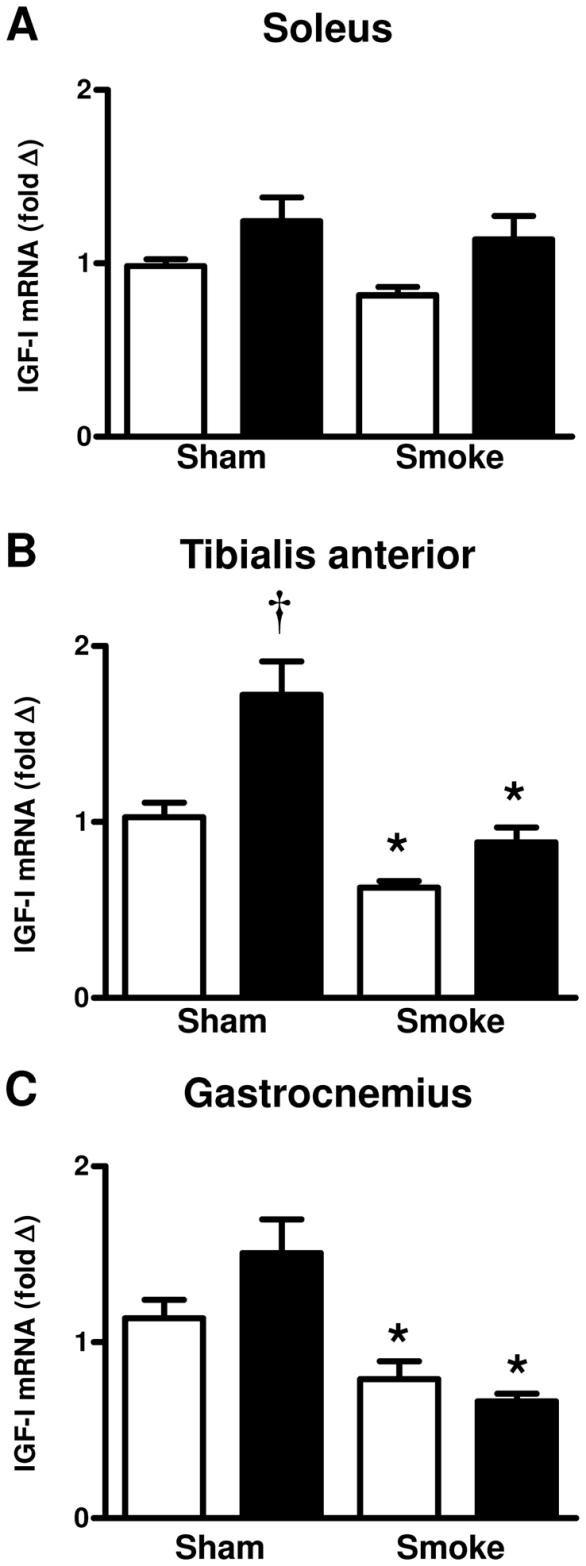
Effect of subchronic cigarette smoke exposure and HFD on IGF-1 mRNA expression in skeletal muscles. Male BALB/c mice were exposed to 4 cigarettes/day, 6 days/week for 7 weeks and the mRNA expression of IGF-I in the soleus (A), tibialis anterior (B) and gastrocnemius (C) skeletal muscles was measured. Mice had access to either standard laboratory chow (□) or a HFD (▪) across the 7 week experimental period. Data are shown as mean±SE and each sample was performed in duplicate (n = 8 per treatment group). Gene expression was normalized to 18S rRNA and expressed as a fold change relative to the Sham and Chow group. Data were analysed by two-way ANOVA and when statistical significance was achieved a *post hoc* Bonferroni test was performed. * P<0.05 significant *post hoc* effect of smoke exposure compared to sham animals on the same diet. † P<0.05 significant *post hoc* effect of HFD compared to chow fed animals.

Cigarette smoke exposure had no effect on IGF-1 mRNA expression in the soleus skeletal muscle (Figure 2A) whereas it had a significant effect on IGF-1 gene expression in both the tibialis anterior and gastrocnemius muscles (Figure 2B and C, P<0.05). Specifically, smoke exposure significantly decreased IGF-1 mRNA expression in both the tibialis anterior and gastrocnemius skeletal muscles of the HFD and chow fed animals compared to sham animals of the same dietary group (P<0.05). The HFD significantly increased IGF-1 mRNA expression in the tibialis anterior compared to chow fed animals (Figure 2B, P<0.05).

**Figure 3:**
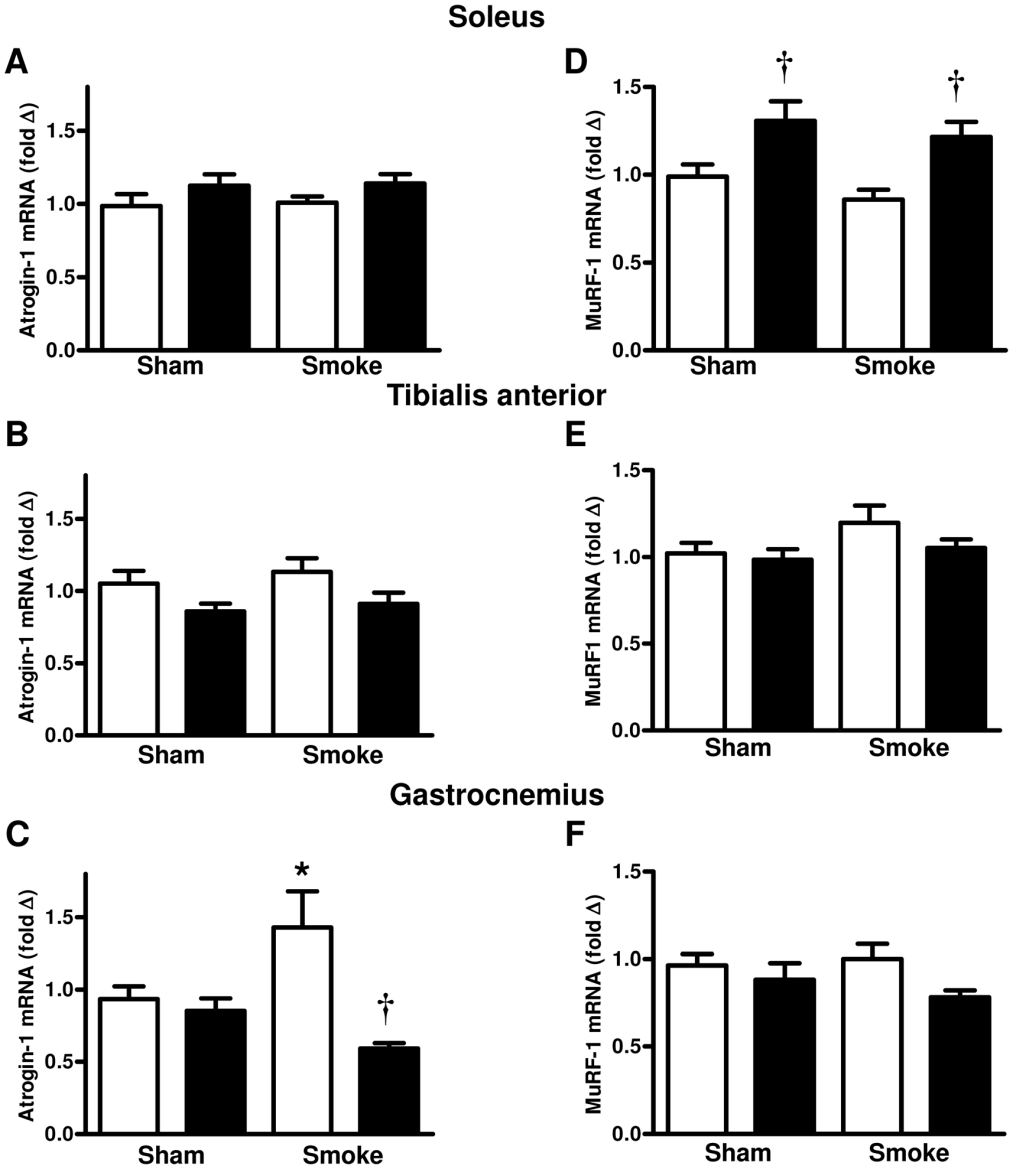
The effect of subchronic cigarette smoke exposure and HFD on the mRNA expression of atrogenes in skeletal muscles. Male BALB/c mice were exposed to 4 cigarettes/day, 6 days/week for 7 weeks and the mRNA expression of atrogin-1 (A-C) and MuRF1 (D-E) in the soleus, tibialis anterior, and gastrocnemius skeletal muscles was determined. Mice had access to either standard laboratory chow (□) or a HFD (▪) across the 7 week experimental period. Data are shown as mean±SE and each sample was performed in duplicate (n = 8 per treatment group). Gene expression was normalized to 18S rRNA and expressed as a fold change relative to the Sham and Chow group. Data were analysed by two-way ANOVA and when statistical significance was achieved a *post hoc* Bonferroni test was performed. * P<0.05 significant *post hoc* effect of smoke exposure compared to sham animals on the same diet. † P<0.05 significant *post hoc* effect of HFD compared to chow fed animals.

The mRNA expression of atrogin-1 was not altered by either the HFD or smoke exposure in the soleus and tibialis anterior skeletal muscles (Figure 3A and B). In the gastrocnemius muscle cigarette smoke exposure significantly increased atrogin-1 mRNA expression of the chow fed animals (Figure 3C, P<0.05). In contrast, the HFD significantly attenuated this smoke-induced increase in atrogin-1 mRNA expression in the gastrocnemius skeletal muscle (Figure 3C, P<0.05). The mRNA expression of MuRF-1 was significantly increased in the soleus skeletal muscle by the HFD in sham and smoke exposed mice (Figure 3D, P<0.05) but was not affected by either cigarette smoke exposure or HFD in the tibialis anterior and the gastrocnemius skeletal muscles (Figure 3E and F).

**Figure 4:**
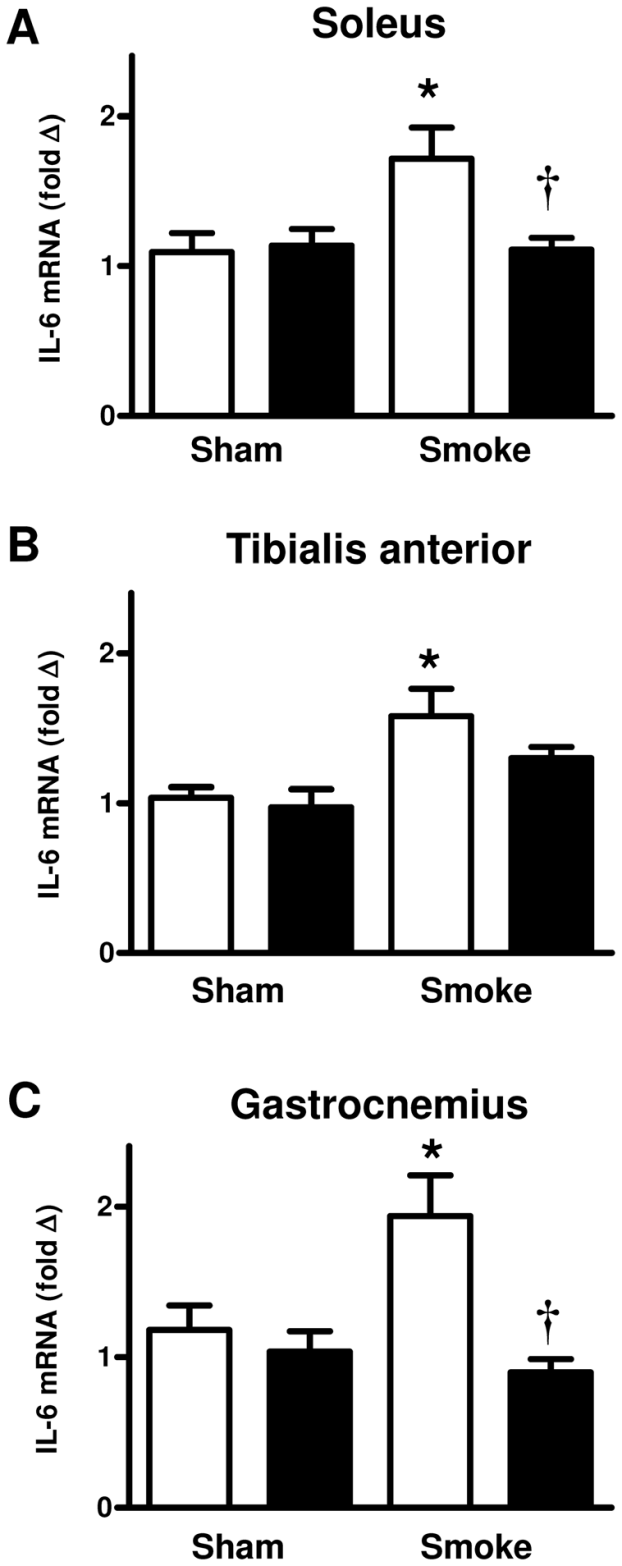
The effect of subchronic cigarette smoke exposure and HFD on IL-6 mRNA expression in skeletal muscles. Male BALB/c mice were exposed to 4 cigarettes/day, 6 days/week for 7 weeks and the mRNA expression of IL-6 in the soleus (A), tibialis anterior (B) and gastrocnemius (C) skeletal muscles was determined. Mice had access to either standard laboratory chow (□) or a HFD (▪) across the 7 week experimental period. Data are shown as mean±SE and each sample was performed in duplicate (n = 8 per treatment group). Gene expression was normalized to 18S rRNA and expressed as a fold change relative to the Sham and Chow group. Data were analysed by two-way ANOVA and when statistical significance was achieved a *post hoc* Bonferroni test was performed. * P<0.05 significant *post hoc* effect of smoke exposure compared to sham animals on the same diet. † P<0.05 significant *post hoc* effect of HFD compared to chow fed animals.

In the chow fed animals, cigarette smoke exposure significantly increased the mRNA expression of IL-6 in the soleus, tibialis anterior and gastrocnemius muscles (Figure 4, P<0.05) and this smoke-induced increase in IL-6 mRNA expression was significantly reduced by the HFD in the soleus and gastrocnemius skeletal muscles (P<0.05). In contrast, IL-6 protein level was not altered by cigarette smoke exposure or the HFD in the gastrocnemius (data not shown). The smoke exposure and HFD had no effect on TNF-α gene expression in the skeletal muscles examined (data not shown) and TNF-α protein level in the gastrocnemius (data not shown).

**Figure 5:**
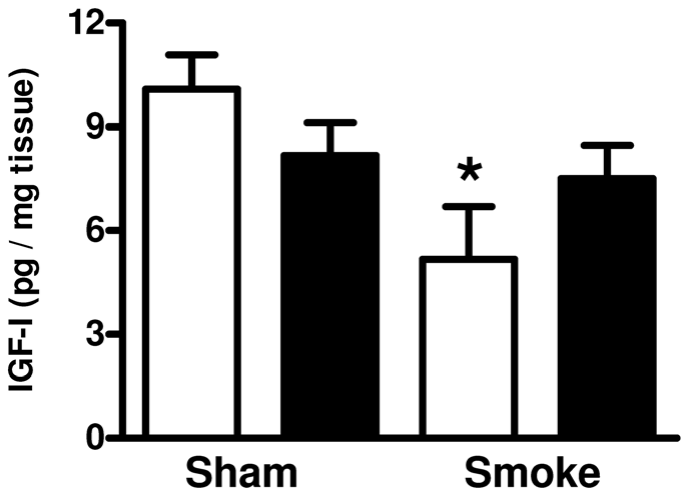
The effect of subchronic cigarette smoke exposure and HFD on IGF-I protein level in the gastrocnemius skeletal muscle. Male BALB/c mice were exposed to 4 cigarettes/day, 6 days/week for 7 weeks and the protein level of IGF-I in the gastrocnemius skeletal muscle was determined. Mice had access to either standard laboratory chow (□) or a HFD (▪) across the 7 week experimental period. Data are shown as mean±SE and each sample was performed in duplicate (n = 8 per treatment group). Data were analysed by two-way ANOVA and when statistical significance was achieved a *post hoc* Bonferroni test was performed. * P<0.05 significant *post hoc* effect of smoke exposure compared to sham animals on the same diet.

While the HFD did not alter IGF-1 protein level in the gastrocnemius skeletal muscle (Figure 5), cigarette smoke exposure significantly decreased IGF-1 protein level (P<0.05) in the chow fed animals (P<0.05).

## Discussion

Tobacco smoking and obesity are the two major causes of preventable morbidity and mortality worldwide and drastically shorten life expectancy but their interaction is poorly understood. To our knowledge this is the first study to examine the effect of a HFD on the lung inflammation and skeletal muscle wasting induced by cigarette smoke exposure in mice. While the pulmonary effects of cigarette smoke exposure have been extensively studied in rodents, the mechanisms responsible for skeletal muscle loss following cigarette smoke exposure remain to be determined. There is accumulating evidence of a causal link between obesity and respiratory diseases like asthma in children, adolescents and adults [39]. Moreover, mice with genetic or diet-induced obesity have innate airway hyperresponsiveness (AHR), increased ozone-induced pulmonary inflammation, altered immune responses to influenza (A/PR8/34) infection and allergen challenge [6], [7], [40]. As there is accumulating evidence that diet-induced obesity can worsen the pulmonary inflammation induced by external noxious stimuli, we hypothesized that the HFD would worsen the lung inflammation and skeletal muscle wasting associated with cigarette smoke exposure in mice. In contrast we found that the pulmonary inflammation and skeletal muscle wasting induced by cigarette smoke exposure was in general not altered by the HFD.

Consistent with our previous reports cigarette smoke exposure [34], [41] significantly induced gene expression of a number of inflammatory cytokines (IL-1β and IL-6) and chemokines (MCP-1, MIP-2, KC and GM-CSF) in lung tissue and significantly increased KC and GM-CSF protein levels in the BALF of both dietary groups. KC is an important neutrophil chemoattractant, whereas GM-CSF promotes neutrophil and macrophage activation and survival. The elevation in KC and GM-CSF protein was concordant with an induction in gene expression in lung tissue of smoke-exposed mice regardless of diet and most likely explains the very large increase in BALF neutrophil numbers. MIP-2 mRNA expression, another important neutrophil chemoattractant, was also increased in the lungs of cigarette smoke-exposed chow and HFD fed animals. The absence of elevated protein levels for several inflammatory cytokines up-regulated at the transcript level most likely reflects differences in transcriptional and translational kinetics but it was not technically feasible to add additional time points to the current study.

Circulating cytokines IL1β, IL-6 and TNF-α are known to promote the secretion of SAA, a pro-inflammatory acute phase protein, from the liver. Human and rodent obesity are associated with chronically elevated levels of SAA, which are reduced following weight loss [42], [43]. The weight gain induced by the HFD in this study was not sufficient to alter SAA levels. Human studies have shown that smoking is associated with increased systemic inflammation [10] and chronic low-grade systemic inflammation is thought to contribute to the skeletal muscle wasting observed in COPD patients [13]–[15], [44], [45]. In the current study, while there was an induction of inflammatory genes in the lung following cigarette smoke exposure systemic inflammation as measured by SAA was not significantly altered. In addition, the level of circulating IL-6 was below the detection limit of the ELISA. Due to a limited volume of sample we could not measure systemic TNF-α level as an alternative marker of systemic inflammation. However it was recently shown that circulating TNF-α was significantly elevated in C57BL/6 mice exposed to cigarette smoke for 6 months but this was not associated with skeletal muscle wasting [46]. In contrast, Tang and colleagues [47] reported both an increase in serum TNF-α and calf skeletal muscle wasting in C57BL/6 mice after 16 weeks of cigarette smoke exposure. Moreover, TNF-α receptor 2 deficiency ameliorated the weight loss and skeletal muscle wasting induced by chronic (18 weeks) cigarette smoke exposure in mice [48]. In the current study, IL-6 mRNA expression was significantly elevated in all skeletal muscles of the cigarette smoke exposed mice and this increase was inhibited by the HFD. The role of local inflammation in skeletal muscle wasting remains controversial. Local IL-6 expression has pro- and anti-inflammatory effects and has been implicated in satellite cell mediated skeletal muscle hypertrophy [49].

In the current study smoke-exposed mice had lower body weights and skeletal muscle wasting of hind limb muscles prior to airspace enlargement. While the inhibitory effect of cigarette smoke on food intake is well established and could account for the reduction in body weight and skeletal muscle masses we have shown previously using long-term pair-feeding (1 and 3 months) that cigarette smoke exposure caused a greater reduction in body weight compared to equivalent food restriction alone [32], [50]. Airspace enlargement was not determined in the current study, however using a very similar protocol, 8 weeks of smoke exposure did not induce changes in airspace in BALB/c mice [51], which typically takes a much longer period of time (6 months) [46] or nose only exposure [52]. As the reduction in body weight and skeletal muscle masses occurred prior to airspace enlargement we suggest that factors(s) in cigarette smoke (reactive oxygen species or nicotine) and/or a secondary effect (hypoxia or low-grade systemic inflammation) of the exposure caused a significant component of the weight loss and skeletal muscle wasting in this model. This was in line with the observations of reduced body weight prior to histological changes in the airways of smoke-exposed guinea pigs and as seen in our study this was in the absence of systemic inflammation [53]. Oxidative stress is a likely candidate given that immediately after a single exposure to cigarette smoke there was a reduction in muscle but not lung glutathione, an important intracellular antioxidant, and increased lipid peroxidation in the plasma [53].

Interestingly, we have reported a similar decrease in body weight and skeletal muscle masses using BALB/c mice seen after much longer periods (16 or 18 weeks) and higher doses of cigarette smoke exposure (10 or 20 cigarettes/day) using C57BL/6 mice [47], [48]. Moreover, we (M.J. Hansen, unpublished findings) and others [46] have shown after 5 and 6 month exposure protocols, respectively, only a small attenuation of weight gain and no difference in skeletal muscle weights in smoke-exposed C57BL/6 mice. BALB/c mice were used in the current study as we have previously found them to be more susceptible than C57BL/6 mice to the pulmonary inflammatory parameters induced by acute (4 days) cigarette smoke exposure [34]. Thus, it appears that as seen with the pulmonary inflammation different mouse strains [34], [54] may have varying susceptibilities to the systemic effects (systemic inflammation, weight loss and skeletal muscle wasting) associated with cigarette smoke exposure and this will form the basis of future studies in our laboratory.

While a lower body mass index has been reported to independently reduce survival of COPD patients [55], in this study, the greater weight gain of the HFD mice was neither protective nor detrimental on the body weight and skeletal muscle masses of cigarette smoke exposed mice. In fact, fat masses were preserved in the HFD fed animals compared to the chow fed mice following cigarette smoke exposure and this may result in serious metabolic health consequences (e.g. insulin insensitivity and cardiovascular outcomes) in the long term [33].

We found a significant reduction in IGF-1 mRNA expression in the tibialis anterior and gastrocnemius skeletal muscles of the smoke-exposed animals. This decrease was reflected in the IGF-1 protein level in the gastrocnemius of chow fed smoke exposed animals. A significant reduction in IGF mRNA expression in the quadriceps of COPD patients compared to healthy aged-matched control subjects has been described [56]. To our knowledge this is the first time changes in IGF-1 levels have been reported in the skeletal muscles and plasma of cigarette smoke-exposed mice. As IGF-1 promotes skeletal muscle hypertrophy by increasing protein synthesis and satellite cell proliferation and differentiation [26], [27], low IGF-1 may promote skeletal muscle wasting in this model.

Petersen and colleagues reported an increase in atrogin-1 but not MuRF1 mRNA expression in the skeletal muscle of smokers compared to never smokers [57]. Similarly, we found that cigarette smoke exposure alone induced atrogin-1 mRNA but not MuRF-1 mRNA expression in the gastrocnemius. In contrast, both E3 ligases were upregulated in the soleus and extensor digitorum longus after a similar period but higher dose of smoke exposure in C57BL/6 mice [47]. Interestingly, in our study and the Petersen study, markers of systemic inflammation (CRP, IL-6, SAA, TNF-α) were not altered, whereas plasma TNF-α levels were markedly elevated in the study by Tang and colleagues in C57BL/6 mice [47]. Thus, systemic inflammation may not be the only mechanism that contributes to the alterations in atrophy markers and skeletal muscle mass observed in smokers [57] and mice in our study. An imbalance in IGF-1 and ubiquitin ligase (atrogin-1) signaling pathways would have important physiological consequences and in the current study these changes appeared early in disease progression.

As skeletal muscle wasting is a powerful predictor of mortality in COPD and is associated with diminished functional status and quality of life [55], it is important to identify factors that exacerbate muscle wasting in order to develop therapeutic interventions. We report for the first time that cigarette smoke induced a down-regulation in total IGF-1 mRNA expression in the tibialis anterior and gastrocnemius skeletal muscles of chow and HFD fed animals. This may contribute to the loss of muscle mass observed in these mice. Therapeutic interventions that increase local IGF-1 level in the skeletal muscle of smoke exposed mice may reverse the muscle loss observed in this model and would avoid the complications of administering IGF-1 systemically. Good animal models will not only aid in the identification of atrophy markers specifically altered by smoke-induced wasting but also allow pharmacological manipulation and evaluation of these potential therapeutic targets to determine whether skeletal muscle mass and function can be improved following prolonged smoke exposure.
